# Elevated serum interferon-α2 associates with activity and flare risk in juvenile-onset systemic lupus erythematosus

**DOI:** 10.1093/rheumatology/keae643

**Published:** 2024-11-26

**Authors:** Valentina Natoli, Yanick J Crow, David P J Hunt, Kukatharmini Tharmaratnam, Andrea L Jorgensen, Michael W Beresford, Christian M Hedrich, Eve M D Smith

**Affiliations:** Department of Women’s and Children’s Health, Institute of Life Course and Medical Sciences, University of Liverpool, UK; Dipartimento di Neuroscienze, Riabilitazione, Oftalmologia, Genetica e Scienze Materno-Infantili, Università degli Studi di Genova, Genoa, Italy; Rheumatology and Autoinflammatory Diseases Unit, IRCCS Istituto Giannina Gaslini, Genoa, Italy; MRC Human Genetics Unit, Institute of Genetics and Cancer, University of Edinburgh, Edinburgh, UK; Laboratory of Neurogenetics and Neuroinflammation, Institut Imagine, Université de Paris, Paris, France; Centre for Clinical Brain Sciences, University of Edinburgh, Edinburgh, UK; UK Dementia Research Institute, University of Edinburgh, Edinburgh, UK; Department of Health Data Science, University of Liverpool Faculty of Health and Life Sciences, Liverpool, UK; Department of Health Data Science, University of Liverpool Faculty of Health and Life Sciences, Liverpool, UK; Department of Women’s and Children’s Health, Institute of Life Course and Medical Sciences, University of Liverpool, UK; Department of Paediatric Rheumatology, Alder Hey Children's NHS Foundation Trust Hospital, Liverpool, UK; Department of Women’s and Children’s Health, Institute of Life Course and Medical Sciences, University of Liverpool, UK; Department of Paediatric Rheumatology, Alder Hey Children's NHS Foundation Trust Hospital, Liverpool, UK; Department of Women’s and Children’s Health, Institute of Life Course and Medical Sciences, University of Liverpool, UK; Department of Paediatric Rheumatology, Alder Hey Children's NHS Foundation Trust Hospital, Liverpool, UK

**Keywords:** jSLE, type I IFN, Simoa, disease activity, biomarker, flare

## Abstract

**Objectives:**

This study investigated serum IFN-α2 as a putative marker of disease activity and predictor of disease flares in juvenile systemic lupus erythematosus (jSLE).

**Methods:**

A total of 222 serum samples were analysed, including 28 healthy controls (HCs), 88 jSLE (159 samples) and 35 juvenile idiopathic arthritis (JIA) patients. IFN-α2 levels were determined using single-molecule array (Simoa). Cross-sectionally, median IFN-α2 levels were compared between patient groups and disease activity state sub-groups. Time to flare was analysed by linear regression. Longitudinally, the ability of the IFN-α2 and other traditional biomarkers (erythrocyte sedimentation rate/ESR, low C3 and anti-dsDNA antibodies) to detect and predict flares was assessed via a generalised linear mixed model.

**Results:**

Cross-sectional analysis showed higher median IFN-α2 levels in the active/intermediate group (median 3185 fg/ml, IQR 48–13 703) compared with the LDAS (571 fg/ml, IQR 57–1310 fg/ml, *P* = 0.04) and remission sub-groups (271 fg/ml, IQR 3–56, *P <*0.001). IFN-α2 was higher in all JSLE patients (median 587 fg/ml, IQR 11–2774) as compared with JIA patients (median 7 fg/ml, IQR 3–236, *P* = 0.0017) and HCs (*P* = 0.017). JSLE patients in remission or LDAS with abnormal IFN-α2 levels had a shorter time to flare over the subsequent six months compared with those with normal IFN-α2 levels (*P* = 0.022). Longitudinally, multivariable analysis demonstrated high IFN-α2 to be the only predictor of an ongoing flare (*P* = 0.028).

**Conclusion:**

Serum IFN-α2 levels associate with disease activity and can predict ongoing and future flares in jSLE. These findings suggest that quantification of IFN-α2 may support risk stratification and disease monitoring in these patients.

Rheumatology key messages

- jSLE patients have higher serum IFN-α2 levels than JIA patients or healthy controls.
- IFN-α2 concentrations associate with disease activity and can predict ongoing and future flares in jSLE.
- Serum IFN-α2 quantification may support risk stratification and disease monitoring in jSLE patients.

## Introduction

Systemic lupus erythematosus (SLE) is a severe, chronic, systemic autoimmune/autoinflammatory disease characterized by intermittent and widespread inflammation [1, 2]. Juvenile-onset SLE (jSLE), defined by symptom onset before the age of 18 years, accounts for ∼15–20% of all SLE patients. When compared with adult-onset disease, jSLE presents generally with a more severe phenotype, higher disease activity and more organ damage, requiring more aggressive immunosuppressive treatments [1, 2]. Overall standardized mortality rates (SMR) are higher in SLE as compared with the general population (SMR 2.2 across all ages), and in patients under the age of 18 years, the SMR is three times higher than in adult-onset SLE (SMR 6.5) [3]. Therefore, timely identification of disease activity and the prediction of flares are critically important to guide therapeutic interventions and to limit organ damage, improving long-term prognosis and ultimately reducing mortality.

Despite the routine use of conventional biomarkers, including anti-double stranded DNA (dsDNA) antibodies, complement levels and erythrocyte sedimentation rates (ESR), to monitor disease activity of SLE patients, none of these have demonstrated sufficient reliability to base therapeutic decision making solely on laboratory parameters [4]. Although the pathogenesis of SLE is complex and still incompletely elucidated [5], the role of type I interferon (IFN), especially IFN-α2, has been established [6].

Direct quantification of IFN is challenging, due to low serum concentrations (even associated with disease flares) that are not detectable by the currently available immunoassays [7, 8]. To overcome this, indirect measurement of IFN pathway activation has been proposed by evaluating the messenger RNA (mRNA) expression of interferon-stimulated genes (ISGs) in peripheral blood cells, known as the IFN signature [9]. Notably, the predominance of type I IFN activation is more pronounced in jSLE than in adult-onset SLE, as 95% of pediatric patients display a pathological type I IFN signature in peripheral blood mononuclear cells, as compared with 50–80% of adult-onset SLE patients [10]. However, to date, the IFN signature has not yet become a common tool in routine laboratory settings, due to the relative complexity of the technique, lack of standardization and its limited availability [7]. A novel ultra-sensitive digital immunoassay known as single-molecule array (Simoa), able to detect extremely low protein concentrations, has overcome those challenges, allowing direct quantification of this cytokine [11]. Studies in adult-onset SLE cohorts demonstrated that the measurement of IFN-α2 with Simoa exhibits similar sensitivity when compared with IFN signatures [12–14]. Notably, in adult-onset SLE, an association between elevated serum IFN-α2 levels and disease activity was demonstrated, and IFN-α2 levels predict future flares in patients who experience clinical remission [12].

Using this highly sensitive digital immunoassay, this study investigated the ability of serum IFN-α2 levels to discern jSLE patients from controls [juvenile idiopathic arthritis (JIA) patients and healthy participants], and jSLE patients with active disease from those in a low disease activity state (LDAS) and/or remission [15–18]. Furthermore, we interrogated whether IFN-α2 levels, alone or combined with conventional laboratory-based biomarkers, could detect ongoing disease flare, predict the risk of flare and estimate time to a subsequent flare.

## Patients, materials and methods

### Study design and participants

This study included jSLE and JIA patients, and healthy participants enrolled in the UK JSLE Cohort Study [19]. Patients fulfilled a minimum of four ACR-1997 classification criteria for SLE and had a minimum of 1-year longitudinal follow-up data recorded (to enable assessment of subsequent flare occurrence at 6 and 12 months post serum IFN-α2 quantification). Although most jSLE patients were recruited at the time of diagnosis, enrolment in the UK JSLE Cohort Study was open to patients at any stage of their disease course. Serum samples were collected between November 2010 and November 2019, after the commencement of jSLE treatment. Data on clinical features, demographics, including ethnicity according to the UK National Census categorizations [20], treatments, standardized disease activity measures [British Isles Lupus Assessment Group (BILAG) 2004 disease activity index [21] and Systemic Lupus Erythematosus Disease Activity Index 2000 (SLEDAI-2k)] were collected alongside each serum sample. Laboratory data collected included anti-dsDNA antibody titres (cut-off value representing anti-dsDNA positivity: 20 IU/ml), complement component 3 levels (C3, cut-off for low: 0.90 g/dl), full blood count (FBC) and erythrocyte sedimentation rate (ESR, normal if below 10 mm/h, mildly to moderately raised if between 10 and 50 mm/h and highly raised if above 50 mm/h).

JSLE patients were divided into four sub-categories including remission [fulfilling any of the four original adult-onset SLE Definition of Remission in SLE (DORIS) 2017 framework remission criteria [16]], LDAS [meeting any of the adult-onset SLE Lupus Clinical Trials Consortium (LCTC LDAS), Asia Pacific Lupus Collaboration Lupus Low Disease Activity State (LLDAS) and Toronto definitions of LDAS [15, 17, 18]]; having intermediate disease active state (SLEDAI-2k score between 5–9); or active disease (SLEDAI-2k score ≥10) (Supplementary Table S1, available at *Rheumatology* online). Disease flare at 6 and 12 months after IFN-α2 measurement was defined according to the BILAG-2004 flare index as a new A or B score in at least one BILAG-2004 domain [22].

Patients with JIA had peripheral blood samples collected alongside basic demographic information and International League Against Rheumatism (ILAR) sub-type classification for JIA [23]. Children <16 years without a past medical history of inflammatory or recent infectious disease were recruited as healthy controls (HC), and peripheral blood samples and demographic information were collected.

### Single-molecule array (Simoa)

Serum IFN-α2 levels, expressed in femtogram per milliliter (fg/ml), were determined by Simoa technology using a commercial kit for IFN-α2 quantification (Quanterix™, Lexington, MA, USA) at the University of Edinburgh following manufacturer’s instructions. The lower limit of detection of this immunoassay was 5 fg/ml, and the upper limit of quantification 52 200 fg/ml. In this study, IFN-α2 values below the lower limit of detection were assigned a standard value equal to 3.53 fg/ml (lower limit of detection/√2) [24]. All serum samples were analysed in duplicate, and mean, median, standard deviation (SD) and coefficient of variation (CV) were calculated. All samples with a CV >20 were excluded. Healthy control IFN-α2 level mean and SD were calculated after removing three extreme outliers with IFN-α2 mean values >1000 fg/ml.

### Statistical analysis

Categorical variables were expressed as numbers (percentage, %), and quantitative variables as the mean±SD or median and interquartile range (IQR), as appropriate. *P*-values <0.05 were considered statistically significant.

Statistical analyses and graphs were performed using R software packages (*dplyr, stats, survival, foreign, glmnet, FSA, gee, lme4, coxme*) version 4.2.0 and GraphPad Prism software version 9.5 (GraphPad Software, San Diego, CA, USA).

#### Cross-sectional analyses

Only one sample per participant was included in the cross-sectional analyses. Where multiple samples were available for a participant, the one with the lowest CV was included. Median IFN-α2 levels were compared between patient groups (HCs, JIA and jSLE patients) and jSLE patient sub-groups (active/intermediate, LDAS or remission). In addition, a sensitivity analysis was conducted to assess IFN-α2 levels across different disease activity states in jSLE patients, incorporating all patient visit data, using a Generalized Linear Mixed Model (GLMM).

Cross-sectional data were utilized for comparing IFN-α2 levels between HCs, JIA and jSLE patients, due to the absence of longitudinal data for the first two groups. A cross-sectional approach was taken when investigating potential differences in IFN-α2 levels among jSLE patient sub-groups defined according to sex, ethnicity, disease activity states and prednisolone dosage, due to the limited availability of jSLE patients with more than one visit (Supplementary Table S2, available at *Rheumatology* online). Student’s *t* test and Mann–Whitney *U* test were used in pairwise comparisons of parametric and nonparametric continuous data, respectively, and Fisher’s exact or χ^2^ test for categorical data. One-way ANOVA followed by Tukey’s *post hoc* test and Kruskal–Wallis followed by Dunn’s *post hoc* tests with Benjamini–Hochberg *P*-value correction method were used when comparing more than two groups in normally distributed and non-normally distributed data, respectively. Differences in time to disease flare within 6 and 12 months for jSLE patients in the cross-sectional analyses were assessed by comparing time to flare between those with normal and abnormal IFN-α2 levels using *t* tests and linear regression. Patients who did not experience a flare within these periods were excluded from the analyses.

#### Longitudinal analyses

As data on serial serum IFN-α2 measurements were available for some jSLE patients (Supplementary Table S2, available at *Rheumatology* online), longitudinal analyses including all available samples were also performed. To investigate the ability of IFN-α2 levels and other traditional jSLE biomarkers (anti-dsDNA antibodies, low C3 levels, ESR) to detect an ongoing flare and to predict a flare at the following visit, univariable and multivariable GLM models were used. Time to flare was assessed using Cox models with cluster effects to account for repeated measures from the same participant. Patients who did not experience a flare during these timeframes were appropriately censored, with their survival time set to the maximum analysis duration of 168 (time to flare within 6 months) and 365 days (time to flare within 12 months). Both univariable analyses, including a covariate to represent each biomarker in turn, and multivariable analyses, including all biomarkers in a single model, were undertaken.

## Results

### Participant characteristics

A total of 291 serum samples from 196 participants were analysed, including 95 jSLE, 52 JIA patients and 49 HCs. After the exclusion of 69 samples with a CV >20 between duplicate analyses, 222 samples were included in the statistical analysis from 88 jSLE (159 samples), 35 JIA patients and 28 HCs (Table 1). A median of one sample per jSLE patient was available (IQR 1–2, range 1–6, Table 1). Supplementary Table S2, available at *Rheumatology* online, illustrates visit/sampling visitation patterns among the jSLE patients studied, showing that 64% had only one visit, while the remaining 36% had more than one visit. Samples from JIA patients and HCs were one per individual. Among all jSLE visits, 71 (45%) were in active or intermediate disease activity state, 65 (41%) in LDAS and 23 (14%) in remission.

**Table 1.** Demographic information of study participants

	All jSLE (*n* = 88)	JIA^a^ (*n* = 35)	HCs (*n* = 28)	*P*-value^b^
Gender, *n* (%)	71 F (81), 17 M (19)	24 F (69), 11 M (31)	23 F (82), 5 M (18)	NS
Age at diagnosis, years (median [IQR])	12.5 [9.5, 14.0]	—	—	NA
Age at sample collection, years (median [IQR])	15.7 [14.0, 18.4]	12.9 [11.1, 14.8]	16.0 [15.7, 16.6]	<0.0001^c^
Ethnicity, *n* (%)				<0.0001^d^
White	35 (44)	34 (100)	24 (86)	
Asian	29 (36)	—	2 (7)	
African/Caribbean	16 (20)	—	2 (7)	
NA	8/88	1/35	—	
Prednisolone dosage, mg (median [IQR])	0.0 [0.0–5.0]	NA	NA	NA
Number of visits per patient [IQR; range]	1 [1, 2; 1–6]	1	1	NA

a JIA sub-types: 11 oligoarticular JIA, 16 polyarticular JIA, 3 juvenile psoriatic arthritis, 5 systemic-onset JIA.

b Comparison of proportions across participant groups (Kruskal–Wallis test).

c Dunn’s *post hoc* tests with Benjamini–Hochberg *P*-value correction method: HCs *vs* JIA *P =*0.0002; JIA *vs* jSLE *P* < 0.0001.

d Dunn’s *post hoc* tests with Benjamini–Hochberg *P*-value correction method: jSLE *vs* JIA *P <*0.0001; jSLE *vs* HCs *P* = 0.007.

F, females; HCs, healthy controls; IQR, interquartile range; JIA, juvenile idiopathic arthritis; jSLE, juvenile systemic lupus erythematosus; LDAS, low disease activity state; M, males; NA, not available/applicable; NS, not significant.

### Cross-sectional analyses

#### Demographics

Comparing demographics of study participants included in the cross-sectional analyses, jSLE patients and HCs were comparable in terms of their age at the time of serum sample collection (median 15.7 years, IQR 14.0–18.4 *vs* median 16.0 years, IQR 15.7–16.6; *P* = 0.7), while jSLE patients were significantly older as compared with JIA patients (median 12.9 years, IQR 11.1–14.8; *P < *0.001). The three groups were comparable in terms of sex distribution (*P* = 0.29). Ethnicity distribution was significantly different among the three study sub-cohorts, with the jSLE group including a higher percentage of individuals of Black African/Caribbean and Asian ethnicity compared with HCs (*P* = 0.007) and JIA patients (*P <* 0.001) (Table 1).

#### Serum IFN-α2 in jSLE, JIA and healthy controls

**Figure 1.**
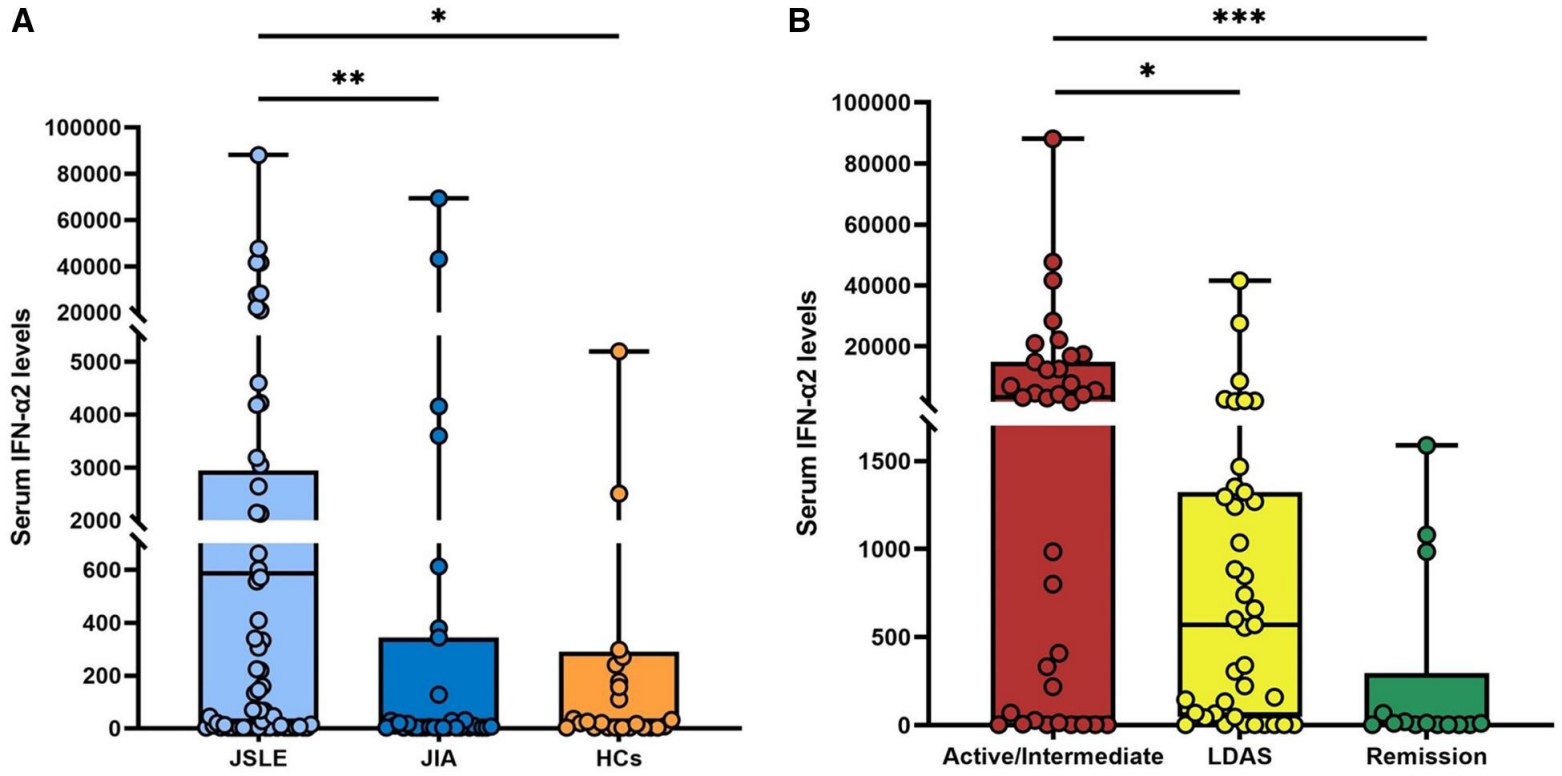
Cross-sectional analysis of serum IFN-α2 in jSLE (according to disease activity), JIA patients and HCs . (**A**) Comparison of serum IFN-α2 levels in jSLE (*n* = 88), JIA (*n* = 35) and HCs (*n* = 28). (**B**) Comparison of serum IFN-α2 levels in jSLE patients (*n* = 88) stratified for disease activity state. Statistically significant post-hoc adjusted *P*-values are displayed. *P ≤0.05; **P ≤0.01; ***P ≤0.001. HCs: healthy controls; IFN-α2: interferon-alpha2; JIA: juvenile idiopathic arthritis; jSLE: juvenile systemic lupus erythematosus; LDAS: low disease activity state

IFN-α2 concentrations were higher in jSLE (median 587 fg/ml, IQR 11–2744) as compared with JIA patients (median 7 fg/ml, IQR 3–236, *P* = 0.0017) and HCs (29 fg/ml, IQR 3–277; *P* = 0.017) (Fig. 1A, Supplementary Table S3, available at *Rheumatology* online), cross-sectionally. No differences in serum IFN-α2 levels were observed between JIA patients and HCs (*P* = 0.581). Serum IFN-α2 levels did not differ between JIA sub-types (oligoarticular, polyarticular, psoriatic, systemic JIA) (Supplementary Table S4, available at *Rheumatology* online). When analysing IFN-α2 levels by sex, no significant differences were found between male and female individuals in any of the patient groups or HCs (Supplementary Table S5, available at *Rheumatology* online).

Patients of Black African/Caribbean ethnicity had higher median serum IFN-α2 levels (1326 fg/ml, IQR 647–7503) compared with White individuals (134 fg/ml, IQR 3–1255, *P* = 0.028), while the levels were similar between Black African/Caribbean and Asians participants (686 fg/ml, IQR 3–2258, *P* = 0.102), and Asian and White patients (*P* = 0.390) (Supplementary Table S6, available at *Rheumatology* online). The distribution of disease activity states did not differ across the three ethnic groups (Supplementary Table S6, available at *Rheumatology* online). Additionally, no differences could be seen in IFN-α2 levels according to prednisolone dosage (Supplementary Table S7, available at *Rheumatology* online).

Cross-sectionally, among jSLE patients, median serum IFN-α2 levels were higher in the combined active/intermediate group (median 3185 fg/ml, IQR 48–13 703) as compared with both the LDAS (571 fg/ml, IQR 57–1310 fg/ml, adjusted *P* = 0.041) and remission (271 fg/ml, IQR 3–56; adjusted *P <* 0.001) sub-groups. Serum IFN-α2 levels in LDAS and remission were comparable (*P* = 0.05), as were median IFN-α2 levels between jSLE patients in remission and HCs (*P* = 0.37) (Fig. 1B, Supplementary Table S8, available at *Rheumatology* online). A significant difference in serum IFN-α2 levels was observed in jSLE patients who flared at 12 months (median 1189 fg/ml, IQR 335–7601) compared with those who did not flare (median 222 fg/ml, IQR 46–1283; *P =* 0.036). However, no significant difference in IFN-α2 levels was found between patients who flared or did not flare at 6 months (*P =* 0.058) (Supplementary Table S9, available at *Rheumatology* online).

#### Abnormal serum IFN-α2 levels predict time to flare

Abnormal IFN-α2 levels were defined as >960 fg/ml. This is equivalent to the average serum IFN-α2 concentration in HC’s plus three standard deviations, in accordance with previous studies [12]. In the cross-sectional cohort of jSLE patients, time to flare (over the following 6 months) was significantly shorter in patients with abnormal IFN-α2 levels (median 91 days to flare, IQR 77–126) compared with those with normal IFN-α2 levels (median 128 days to flare, IQR 126–137, *P =* 0.022, Table 2). Within 12 months after IFN-α2 measurement, an equal proportion (50%) of those with abnormal and normal IFN-α2 levels experienced a disease flare (*P =* 1), with no significant differences in the time to flare between groups (*P =* 0.2, Table 2).

**Table 2.** Cross sectional analysis comparing time to flare (within 6 and 12 months) in jSLE patients in remission or LDAS with normal and abnormal IFN-α2 levels

Outcome variable	All patients in remission or LDAS (*n* = 53)	Patients in remission or LDAS with abnormal IFN-α2 (*n* = 17)	Patients in remission or LDAS with normal IFN-α2 (*n* = 36)	*P-*value	*t* test *P*-value
**Time to flare within 6 months of IFN-α2 quantification (days)**					
Mean (SD)	120.9 (31.1)	98.0 (26.6)	137.3 (23.4)	**0.022** ^a^ (−39.29 days)	**0.029**
Median (Q1, Q3)	126.0 (101.5, 131.0)	91.0 (77.0, 126.0)	128.0 (126.0, 137.3)		
Min, Max	70.0, 168.0	70.0, 126.0	150.0, 168.0		
Flare, *n* (%)	12 (32)	5 (36)	7 (29)	0.728^b^	NA
No flare, *n* (%)	26 (68)	9 (64)	17 (71)		
NA, *n*	15	3	12		
**Time to flare within 12 months of IFN-α2 quantification (days)**					
Mean (SD)	157.4 (59.9)	134.0 (65.3)	171.1 (54.7)	0.201^a^ (−37.08 days)	0.232
Median (Q1, Q3)	140.0 (126.0, 196.0)	126.0 (84.0, 171.5)	168.0 (127.5, 196.0)		
Min, Max	70.0, 308.0	70.0, 231.0	105.0, 308.0		
Flare, *n* (%)	19 (50)	7 (50)	12 (50)	1.000^b^	NA
No flare, *n* (%)	19 (50)	7 (50)	12 (50)		
NA, *n*	15	3	12		

a Linear regression model.

b Fisher’s exact test. N=number of patients/samples. IFN-α2, interferon-alfa2; jSLE, juvenile systemic lupus erythematosus; LDAS, low disease activity state; Max, maximum; Min, minimum; NA, not available; Q1, first quartile; Q3, third quartile; SD, standard deviation.

Bold values in the *P*-value columns indicate statistically significant results (*P* < 0.05).

### Longitudinal analyses

Longitudinal analyses included data from 88 jSLE patients with a total of 159 serum IFN-α2 measurements over the follow-up visits. The distribution of visits per patient is detailed in Supplementary Table S2, available at *Rheumatology* online.

#### Comparison of serum IFN-α2 levels in jSLE patients stratified for disease activity state

Using GLM modelling, we analysed serum IFN-α2 levels across disease activity states in jSLE patients, incorporating measurement from all 159 samples. Patients with active or intermediate disease activity (serving as reference group) displayed numerically higher median serum IFN-α2 levels (median 1583 fg/ml, IQR 100–7850), compared with patients in LDAS (median 228 fg/ml, IQR 3–1278) or remission (median 11 fg/ml, IQR 3–291), though these reductions did not reach statistical significance (*P =* 0.099 for LDAS; *P =* 0.089 for remission) (Supplementary Table 10, available at *Rheumatology* online).

#### Comparison of time to flare in jSLE patients with normal and abnormal IFN-α2 levels

Cox proportional hazards with cluster effects modelling incorporating data from all longitudinal jSLE patient visits irrespective of disease activity state was used to compare time to flare (within 6 and 12 months) in jSLE patients with normal and abnormal IFN-α2 levels. Patients with abnormal IFN-α2 levels exhibited a significantly higher risk of experiencing a shorter time to flare within 6 months (median 91 days, IQR 70–126) as compared with those with normal levels (median 128 days, IQR 119–168, *P =* 0.041), with a hazard ratio (HR) of 2.1 (95% CI 1.0–4.2, Supplementary Table S11, available at *Rheumatology* online).

#### Ability of serum IFN-α2 and traditional biomarkers to detect an ongoing disease flare and predict a future flare in jSLE

Incorporating data from all longitudinal jSLE patient visits, the ability to detect an ongoing flare and to predict a flare at the following visit according to abnormal serum IFN-α2 levels and traditional laboratory biomarkers (positive anti-dsDNA autoantibodies, low C3 levels and raised ESR) was investigated using GLMM. Univariable GLMM analyses demonstrated that abnormal IFN-α2 was the only marker able to detect ongoing disease flares at the time of the visit (OR 4.80 [95% CI 1.59–14.54], *P =* 0.005). Within the multivariable analysis, elevated serum IFN-α2 continued to be the only variable associated with being currently in a flare (OR 3.84 [95% CI 1.15–12.80], *P =* 0.028) (Table 3).

**Table 3.** Longitudinal analyses investigating the ability of IFN-α2 and standard clinical biomarkers to detect an ongoing disease flare in jSLE

	GLMM OR (95% CI)	GLMM *P*-value
**Univariable analyses**		
**IFN-α2 levels, fg/mL (*n* = 159)**		
Normal (*n* = 93)		
Abnormal (*n* = 66)	4.80 (1.59, 14.54)	**0.005**
**Anti-dsDNA abs, IU/mL (*n* = 155)**		
<20 (*n* = 62)		
≥20 (*n* = 93)	1.17 (0.42, 3.26)	0.765
**C3 levels, g/dL (*n* = 158)**		
≥0.90 (*n* = 89)		
<0.90 (*n* = 69)	0.93 (0.35, 2.50)	0.894
**ESR, mm/h (*n* = 157)**		
Normal <10 (*n* = 69)		
Mildly to mod raised 10–50 (*n* = 64)	1.24 (0.44, 3.49)	0.676
High >50 (*n* = 24)	3.78 (0.72, 19.78)	0.115
**Multivariable analysis (*n* = 153)**		
**IFN-α2 levels, fg/mL**		
Normal		
Abnormal	3.84 (1.15–12.80)	**0.028**
**Anti-dsDNA abs, IU/mL**		
<20		
≥20	0.82 (0.24–2.81)	0.751
**C3 levels, g/dL**		
≥0.90		
<0.90	0.79 (0.26-2.42)	0.678
**ESR, mm/h**		
Normal <10		
Mildly to mod raised 10–50	0.90 (0.28, 2.95)	0.865
High >50	2.98 (0.43, 20.48)	0.266

N = number of measurements for which laboratory data were available. Bold values in the *P*-value columns indicate statistically significant results (*P* < 0.05).

anti-dsDNA abs: anti-double stranded DNA antibodies; CI: confidence interval; ESR: erythrocyte sedimentation rate; g/dL: grams per deciliter; GLMM: generalised linear mixed model; IFN-α2: interferon-alfa2; IU/mL: international units per milliliter; jSLE: juvenile systemic lupus erythematosus; mm/h: millimeters per h; OR: odd ratio; SD: standard deviation.

In univariable analyses, abnormal serum IFN-α2 levels predicted subsequent disease flares, with an OR of 2.57 (95% CI 1.02–6.46, *P =* 0.045), suggesting a potential association. However, this association was not confirmed in the multivariable analysis, where abnormal serum IFN-α2 levels did not reach statistical significance for flare prediction, showing an OR of 2.40 (95% CI 0.87–6.62, *P =* 0.089). None of the traditional laboratory biomarkers displayed an association with the risk of flare at the next visit (Table 4). No biomarkers demonstrated a significant ability to predict time to flare analyses utilizing longitudinal data (Supplementary Table S12, available at *Rheumatology* online).

**Table 4.** Longitudinal analyses investigating the ability of IFN-α2 and standard clinical biomarkers to predict jSLE flare risk at the following visit

	GLMM OR (95% CI)	GLMM *P*-value
**Univariable analyses**		
**IFN-α2 levels, fg/mL (*n* = 150)**		
Normal (*n* = 90)		
Abnormal (*n* = 60)	2.57 (1.02, 6.46)	**0.045**
**Anti-dsDNA abs, IU/mL (*n* = 147)**		
<20 (*n* = 89)		
≥20 (*n* = 58)	1.34 (0.52, 3.45)	0.540
**C3 levels, g/dL (*n* = 149)**		
≥0.90 (82)		
<0.90 (67)	1.13 (0.47, 2.75)	0.778
**ESR, mm/h (*n* = 148)**		
Normal <10 (*n* = 68)		
Mildly to mod raised 10–50 (*n* = 57)	0.76 (0.29, 1.96)	0.573
High >50 (*n* = 23)	2.07 (0.51, 8.33)	0.306
**Multivariable analysis (*n* = 145)**		
**IFN-α2 levels, fg/mL**		
Normal		
Abnormal	2.40 (0.87, 6.62)	0.089
**Anti-dsDNA abs, IU/mL**		
<20		
≥20	1.12 (0.37, 3.42)	0.842
**C3 levels, g/dL**		
≥0.90		
<0.90	0.96 (0.37, 2.51)	0.938
**ESR, mm/h**		
Normal <10		
Mildly to mod raised 10–50	0.54 (0.19, 1.59)	0.265
High >50	1.37 (0.26, 7.18)	0.711

N = number of measurements for which laboratory data were available. Bold values in the *P*-value columns indicate statistically significant results (*P* < 0.05).

anti-dsDNA abs: anti-double stranded DNA antibodies; CI: confidence interval; ESR: erythrocyte sedimentation rate; fg/mL: femtograms per milliliter; g/dL: grams per deciliter; GLMM: generalised linear mixed model; IFN-α2: interferon-alfa2; IU/mL: international units per milliliter; jSLE: juvenile systemic lupus erythematosus; mm/h: millimeters per h; OR: odd ratio; SD: standard deviation.

## Discussion

This is the largest study to date directly assessing IFN-α2 levels in jSLE, using the Simoa digital immunoassay. It is unique by including both an inflammatory control group (JIA patients) and HCs. The study demonstrated that serum IFN-α2 levels are elevated in jSLE patients as compared with JIA patients and HCs. Furthermore, serum IFN-α2 levels associate with disease activity states in jSLE patients and predict future flares.

The observation that jSLE patients have higher IFN-α2 levels than JIA patients and HCs, and that those levels are linked to disease activity and flare occurrence, aligns with the established role of this cytokine in the pathogenesis of SLE [6]. This is further corroborated by the efficacy of inhibition of type I IFN signalling in jSLE patients, including Janus kinase (JAK) inhibitors [25], IFN antibodies (e.g. rontalizumab, sifalimumab) [26, 27] and type I IFN receptor blockers (e.g. anifrolumab) [28]. Increased type I IFN expression in SLE involves a complex interplay between genetic contributors and immune responses to tissue damage. Genetic variants affecting the clearance of cytoplasmic nucleic acids and apoptotic material or enhancing the activation of cytoplasmic acid sensors contribute to type I IFN production [6, 29]. In this context, increased genetic burden in the paediatric population may (at least partially) explain the more pronounced IFN expression observed in jSLE, potentially contributing to the more severe clinical phenotype in children when compared with adult patients [30]. Additionally, tissue damage secondary to several stimuli (e.g. infections, ultraviolet radiation, mechanical stress, etc), results in the accumulation of cellular debris and nuclear material in the extracellular compartment, activation of TLR3/7 pathways, and, ultimately, IFN production [31, 32].

Notably, within this jSLE cohort, serum IFN-α2 levels were significantly higher in patients of African/Caribbean descent compared with White and Asian participants, regardless of disease activity state. The association between elevated serum IFN-α2 levels and African/Caribbean ethnicity has previously been reported [33]. This could be related to genetic contributing factors and/or the more severe phenotype observed in these patients, with more tissue damage and higher mortality [34]. In line with previous reports demonstrating limited ability of glucocorticoids to influence the type I IFN pathway [35, 36], we found no significant association between IFN-α2 levels and glucocorticoid treatment dosage.

In adult-onset SLE, elevated serum IFN-α2 levels measured by Simoa associate with high disease activity and may represent an independent predictive biomarker of disease flare in patients clinically in remission [12, 14]. Notably, Simoa-assessed serum IFN-α2 levels outperformed anti-dsDNA antibodies in identifying active disease and predicting future flares in patients in remission [12, 14]. In both pediatric and adult-onset patients, recent studies showed that IFN-α2 levels quantified with Simoa and IFN signatures are equally able to characterize specific disease activity states [37, 38]. However, the majority of previous studies have predominantly investigated the ISG score as a means to assess IFN-pathway activation (rather than directly quantifying serum IFN-α2 levels), despite its use in clinical practice not being validated [13]. Studies investigating the correlation between ISG score or surrogate markers of type I IFN (e.g. CXCL10, galectin-9) and disease activity in SLE patients have yielded inconsistent results, with some showing a correlation with ISG score or IFN surrogates [44] and others finding no such association [39, 40]. This may be partly because IFN scores are based on genes that could be induced not only by different IFNs (IFN-α, IFN-β and IFN-γ subtypes) but also by additional cytokines, such as TNF-α, thus limiting its specificity [19, 41]. While the Simoa platform is initially moderately expensive, its high sensitivity, specificity and low per-sample cost make it cost-effective for large-scale studies [42]. In this context, direct quantification of serum IFN-α2 levels using Simoa may represent an additional valuable, cost-effective high-throughput tool to detect and predict disease flares and identify patients who may benefit from targeted therapies.

International recommendations for both jSLE and adult-onset SLE [43, 44] support the implementation of a T2T approach for patient management. Indeed, observational studies demonstrate that remission or LDAS target attainment associate with reduced damage accrual and flare frequency, glucocorticoid sparing, improved quality of life and survival [45, 46]. However, because of the aforementioned difficulties in measuring disease activity, current T2T clinical targets may not fully capture ‘biological’ disease activity. Thus, incorporating more objective measures that reflect subclinical systemic inflammation, such as serial IFN-α2 monitoring, may also prove valuable to predict future flares. However, validation of these reported findings in larger, independent and prospective cohorts is necessary.

While this study indicates potential for IFN-α2 as a measure of disease activity and predictor of flares, this study has limitations. Despite representing a relatively large cohort for jSLE, the sample size remains limited in comparison to previous studies in adult-onset SLE cohorts. Although multiple visits per patient (1–6 visits) were available, the majority of the jSLE cohort had only one recorded visit, reducing statistical power and impacting use of more complex models utilizing longitudinal data. In future studies it would be useful to measure IFN-α2 levels at diagnosis (pre-treatment) and to track these longitudinally. Furthermore, a relatively high variation of IFN-α2 results in some experimental duplicates (23% of samples) was observed using Simoa. This was more marked for values near the lower limit of detection (5 fg/ml), impacting especially on IFN-α2 values observed in patients with JIA and in HCs. Auto-antibodies directed against IFN-α2 have been shown to be present in up to a quarter of SLE patients [47] and could theoretically interfere with quantification of IFN-α2 via the Simoa assay [47], potentially limiting the reliability of this assay in some patients. Other potential limitations of the Simoa assay could include antibody specificity, cross-reactivity, standardization and limitations in the assays dynamic range with loss of quantitative accuracy for samples with very high IFN-α2 levels. Thus, additional work is required to further improve IFN-α2 quantification prior to its introduction into clinical practice. Furthermore, data on disease activity in JIA patients were not available. Therefore, we cannot exclude that the differences observed between jSLE and JIA may also be (partially) due to variable disease activity states between disease groups. Finally, we were not able to assess the potential use of serum IFN-α2 as a predictor of disease onset in children with suspected jSLE, as samples from individuals without overt jSLE were not available. Further studies on longitudinally followed cohorts of healthy individuals may help to define if increased IFN-α2 levels may be observed before disease onset, similarly to what has been described for autoantibodies in adult-onset SLE patients [48].

## Conclusions

Observations from this study suggest that quantification of IFN-α2 may support monitoring of disease activity in jSLE and predicting future flares. Further research, including larger independent cohorts from prospective studies is warranted to confirm findings and, possibly, to evaluate if IFN-α2 is able to predict disease onset in individuals with suspected early disease. Additionally, establishing whether this cost-effective, high-throughput and sensitive digital immunoassay could support T2T strategies in jSLE is a crucial next step.

## Supplementary material

Supplementary material is available at *Rheumatology* online.

## Supplementary Material

keae643_Supplementary_Data

## Data Availability

Data are available from the corresponding author upon reasonable request.

## References

[1] Ambrose N , Morgan TA, Galloway J et al; UK JSLE Study Group. Differences in disease phenotype and severity in SLE across age groups. Lupus 2016;25:1542–50. 27147622 10.1177/0961203316644333 PMC5089221

[2] Smith EMD , Lythgoe H, Midgley A, Beresford MW, Hedrich CM. Juvenile-onset systemic lupus erythematosus: update on clinical presentation, pathophysiology and treatment options. Clin Immunol 2019;209:108274. 31678365 10.1016/j.clim.2019.108274

[3] Chen Y-M , Lin C-H, Chen H-H et al Onset age affects mortality and renal outcome of female systemic lupus erythematosus patients: a nationwide population-based study in Taiwan. Rheumatology 2014;53:180–5. 24136069 10.1093/rheumatology/ket330

[4] Floris A , Piga M, Cauli A, Mathieu A. Predictors of flares in Systemic Lupus Erythematosus: preventive therapeutic intervention based on serial anti-dsDNA antibodies assessment. Analysis of a monocentric cohort and literature review. Autoimmun Rev 2016;15:656–63. 26921641 10.1016/j.autrev.2016.02.019

[5] Hedrich CM , Smith EMD, Beresford MW. Juvenile-onset systemic lupus erythematosus (jSLE) – pathophysiological concepts and treatment options. Best Practice & Research Clinical Rheumatology 2017;31:488–504. 29773269 10.1016/j.berh.2018.02.001

[6] Crow MK. Type I interferon in the pathogenesis of lupus. J Immunol 2014;192:5459–68. 24907379 10.4049/jimmunol.1002795 PMC4083591

[7] Lamot L , Niemietz I, Brown KL. Methods for type I interferon detection and their relevance for clinical utility and improved understanding of rheumatic diseases. Clin Exp Rheumatol 2019;37:1077–83. 30943143

[8] Brkic Z , Versnel MA. Type I IFN signature in primary Sjögren’s syndrome patients. Expert Rev Clin Immunol 2014;10:457–67. 24450331 10.1586/1744666X.2014.876364

[9] Obermoser G , Pascual V. The interferon-α signature of systemic lupus erythematosus. Lupus 2010;19:1012–9. 20693194 10.1177/0961203310371161 PMC3658279

[10] Chiche L , Jourde-Chiche N, Whalen E et al Modular transcriptional repertoire analyses of adults with systemic lupus erythematosus reveal distinct type I and type II interferon signatures: modular interferon signatures and systemic lupus erythematosus. Arthritis Rheumatol 2014;66:1583–95. 24644022 10.1002/art.38628 PMC4157826

[11] Rissin DM , Kan CW, Campbell TG et al Single-molecule enzyme-linked immunosorbent assay detects serum proteins at subfemtomolar concentrations. Nat Biotechnol 2010;28:595–9. 20495550 10.1038/nbt.1641 PMC2919230

[12] Mathian A , Mouries-Martin S, Dorgham K et al Ultrasensitive serum interferon-α quantification during SLE remission identifies patients at risk for relapse. Ann Rheum Dis 2019;78:1669–76. 31570366 10.1136/annrheumdis-2019-215571

[13] Rodero MP , Decalf J, Bondet V et al Detection of interferon alpha protein reveals differential levels and cellular sources in disease. J Exp Med 2017;214:1547–55. 28420733 10.1084/jem.20161451 PMC5413335

[14] Mathian A , Mouries-Martin S, Dorgham K et al Monitoring disease activity in systemic lupus erythematosus with single‐molecule array digital enzyme‐linked immunosorbent assay quantification of serum interferon‐α. Arthritis Rheumatol 2019;71:756–65. 30507062 10.1002/art.40792

[15] Polachek A , Gladman DD, Su J, Urowitz MB. Defining low disease activity in systemic lupus erythematosus: low disease activity in SLE. Arthritis Care Res 2017;69:997–1003. 10.1002/acr.23109 27696791

[16] van Vollenhoven RF , Bertsias G, Doria A et al 2021 DORIS definition of remission in SLE: final recommendations from an international task force. 9. 10.1136/lupus-2021-000538 PMC8614136 34819388

[17] Ko K , Levine AB, Griffin R et al Baseline predictors of remission and low disease activity using recently defined international criteria in a multi-center lupus registry cohort [abstract]. Arthritis Rheumatol 2015;67(Suppl 10).

[18] Franklyn K , Lau CS, Navarra SV et al; Asia-Pacific Lupus Collaboration. Definition and initial validation of a Lupus Low Disease Activity State (LLDAS). Ann Rheum Dis 2016;75:1615–21. Sep 26458737 10.1136/annrheumdis-2015-207726

[19] Watson L , Leone V, Pilkington C et al; UK Juvenile-Onset Systemic Lupus Erythematosus Study Group. Disease activity, severity, and damage in the UK Juvenile-Onset Systemic Lupus Erythematosus Cohort. Arthritis Rheum 2012;64:2356–65. 22294381 10.1002/art.34410

[20] UK census [Internet]. Available from: Office of National Statistics (UK). 2001 Census. URL: http://www. statistics.gov.uk/hub/people-places/people/identity/index.html

[21] Marks SD , Pilkington C, Woo P, Dillon MJ. The use of the British Isles Lupus Assessment Group (BILAG) index as a valid tool in assessing disease activity in childhood-onset systemic lupus erythematosus. Rheumatology 2004;43:1186–9. 15226518 10.1093/rheumatology/keh284

[22] Gordon C , Sutcliffe N, Skan J, Stoll T, Isenberg DA. Definition and treatment of lupus flares measured by the BILAG index. Rheumatology (Oxford) 2003;42:1372–9. 12810926 10.1093/rheumatology/keg382

[23] Petty RE , Southwood TR, Manners P et al; International League of Associations for Rheumatology. International League of Associations for Rheumatology classification of juvenile idiopathic arthritis: second revision, Edmonton, 2001. J Rheumatol 2004;31:390–2. 14760812

[24] Ogden TL. Handling results below the level of detection. Ann Occup Hyg 2010;54:255–6. 20067938 10.1093/annhyg/mep099

[25] Mok CC. The Jakinibs in systemic lupus erythematosus: progress and prospects. Expert Opin Investig Drugs 2019;28:85–92. 10.1080/13543784.2019.1551358 30462559

[26] Khamashta M , Merrill JT, Werth VP et al; CD1067 Study Investigators. Sifalimumab, an anti-interferon-α monoclonal antibody, in moderate to severe systemic lupus erythematosus: a randomised, double-blind, placebo-controlled study. Ann Rheum Dis 2016;75:1909–16. 27009916 10.1136/annrheumdis-2015-208562 PMC5099191

[27] Kalunian KC , Merrill JT, Maciuca R et al A Phase II study of the efficacy and safety of rontalizumab (rhuMAb interferon-α) in patients with systemic lupus erythematosus (ROSE). Ann Rheum Dis 2016;75:196–202. 26038091 10.1136/annrheumdis-2014-206090

[28] Morand EF , Furie R, Tanaka Y et al; TULIP-2 Trial Investigators. Trial of Anifrolumab in Active Systemic Lupus Erythematosus. N Engl J Med 2020;382:211–21. 31851795 10.1056/NEJMoa1912196

[29] Bronson PG , Chaivorapol C, Ortmann W, Behrens TW, Graham RR. The genetics of type I interferon in systemic lupus erythematosus. Curr Opin Immunol 2012;24:530–7. 22889593 10.1016/j.coi.2012.07.008

[30] Reid S , Alexsson A, Frodlund M et al High genetic risk score is associated with early disease onset, damage accrual and decreased survival in systemic lupus erythematosus. Ann Rheum Dis 2020;79:363–9. 31826855 10.1136/annrheumdis-2019-216227 PMC7034364

[31] Kono DH , Baccala R, Theofilopoulos AN. TLRs and interferons: a central paradigm in autoimmunity. Curr Opin Immunol 2013;25:720–7. 24246388 10.1016/j.coi.2013.10.006 PMC4309276

[32] Gallucci S , Meka S, Gamero AM. Abnormalities of the type I interferon signaling pathway in lupus autoimmunity. Cytokine 2021;146:155633. 34340046 10.1016/j.cyto.2021.155633 PMC8475157

[33] Ko K , Franek BS, Marion M et al Genetic ancestry, serum interferon-α activity, and autoantibodies in systemic lupus erythematosus. J Rheumatol 2012;39:1238–40. 22505704 10.3899/jrheum.111467 PMC3381952

[34] Massias JS , Smith EM, Al-Abadi E et al Clinical and laboratory phenotypes in juvenile-onset Systemic Lupus Erythematosus across ethnicities in the UK. Lupus 2021;30:597–607. 33413005 10.1177/0961203320984251 PMC7967896

[35] Northcott M , Gearing LJ, Nim HT et al Glucocorticoid gene signatures in systemic lupus erythematosus and the effects of type I interferon: a cross-sectional and in-vitro study. Lancet Rheumatol 2021;3:e357–70. 38279391 10.1016/S2665-9913(21)00006-0

[36] Guiducci C , Gong M, Xu Z et al TLR recognition of self nucleic acids hampers glucocorticoid activity in lupus. Nature 2010;465:937–41. 20559388 10.1038/nature09102 PMC2964153

[37] Chasset F , Mathian A, Dorgham K et al Serum interferon-α levels and IFN type I-stimulated genes score perform equally to assess systemic lupus erythematosus disease activity. Ann Rheum Dis 2022;81:901–3. 35091421 10.1136/annrheumdis-2021-221835

[38] Wahadat MJ , Qi H, van Helden-Meeuwsen CG et al Serum IFNα2 levels are associated with disease activity and outperform IFN-I gene signature in a longitudinal childhood-onset SLE cohort. Rheumatology. 2022 2023;62:2872–9. 10.1093/rheumatology/keac698 PMC10393436 36515466

[39] Enocsson H , Wetterö J, Eloranta M-L et al Comparison of surrogate markers of the type I interferon response and their ability to mirror disease activity in systemic lupus erythematosus. Front Immunol 2021;12:688753. 34276678 10.3389/fimmu.2021.688753 PMC8278235

[40] Northcott M , Jones S, Koelmeyer R et al Type 1 interferon status in systemic lupus erythematosus: a longitudinal analysis. Lupus Sci Med 2022;9:e000625. 35197305 10.1136/lupus-2021-000625 PMC8867321

[41] Wang W , Xu L, Brandsma JH et al Convergent transcription of interferon-stimulated genes by TNF-α and IFN-α augments antiviral activity against HCV and HEV. Sci Rep 2016;6:25482. 27150018 10.1038/srep25482 PMC4858707

[42] Mora J , Given Chunyk A, Dysinger M et al Next generation ligand binding assays-review of emerging technologies’ capabilities to enhance throughput and multiplexing. AAPS J 2014;16:1175–84. 25193269 10.1208/s12248-014-9660-1 PMC4389750

[43] Smith EMD , Aggarwal A, Ainsworth J et al; cSLE T2T International Task Force. Towards development of treat to target (T2T) in childhood-onset systemic lupus erythematosus: PReS-endorsed overarching principles and points-to-consider from an international task force. Ann Rheum Dis 2023;82:788–98. 36627168 10.1136/ard-2022-223328 PMC10314055

[44] van Vollenhoven RF , Mosca M, Bertsias G et al Treat-to-target in systemic lupus erythematosus: recommendations from an international task force. Ann Rheum Dis 2014;73:958–67. 24739325 10.1136/annrheumdis-2013-205139

[45] Fanouriakis A , Adamichou C, Koutsoviti S et al Low disease activity—irrespective of serologic status at baseline—associated with reduction of corticosteroid dose and number of flares in patients with systemic lupus erythematosus treated with belimumab: a real-life observational study. Semin Arthritis Rheum 2018;48:467–74. 29555348 10.1016/j.semarthrit.2018.02.014

[46] Golder V , Tsang-A-Sjoe MWP. Treatment targets in SLE: remission and low disease activity state. Rheumatology 2020;59:v19–28. 33280016 10.1093/rheumatology/keaa420 PMC7719036

[47] Mathian A , Breillat P, Dorgham K et al Lower disease activity but higher risk of severe COVID-19 and herpes zoster in patients with systemic lupus erythematosus with pre-existing autoantibodies neutralising IFN-α. Ann Rheum Dis 2022;81:1695–703. 35973806 10.1136/ard-2022-222549

[48] Arbuckle MR , McClain MT, Rubertone MV et al Development of autoantibodies before the clinical onset of systemic lupus erythematosus. N Engl J Med 2003;349:1526–33. 14561795 10.1056/NEJMoa021933

