# Supplementary material for: Elevated serum interferon-α2 associates with activity and flare risk in Juvenile-onset Systemic Lupus Erythematosus
Source: Rheumatology (Oxford). Author manuscript; Available in PMC 2025 Jun 1. (PMC7617100; doi:10.1093/rheumatology/keae643)
Supplement: Supplementary file 1 [file EMS201638-supplement-Supplementary_Materials.docx]

**Supplementary material** 

**Supplementary Table S1. Remission (DORIS 2017 definitions), LDAS, intermediate, active disease activity state definitions in SLE**

|  | **Complete remission OFF treatment** | **Complete remission ON treatment** | **Clinical remission OFF treatment** | **Clinical remission ON treatment** | **APLC LLDAS** | **Adapt LCTC LDAS**(17) | **Toronto LDA**(15) | **Intermediate activity state** | **Active disease** |
| --- | --- | --- | --- | --- | --- | --- | --- | --- | --- |
| **Disease activity** | SLEDAI-2K=0 OR BILAG – all domains D or E | SLEDAI-2K=0 OR BILAG – all domains D or E | cSLEDAI-2K=0 OR BILAG – all domains D or E | cSLEDAI -2K=0 OR BILAG – all domains D or E | SLEDAI-2k ≤4, no major organs^a^, no new features | SLEDAI-2k ≤4 | SLEDAI-2k <3^b^ | SLEDAI-2k 5-9 | SLEDAI-2k ≥10 |
| **PGA** | <0.5 | <0.5 | <0.5 | <0.5 | ≤1 | <1 | - | - | - |
| **Serology** | Negative anti-dsDNA antibodies, normal C3/C4 | Negative anti-dsDNA antibodies, normal C3/C4 | anti-dsDNA antibodies, low C3 or C4 allowed | High anti-dsDNA antibodies, low C3 or C4 allowed | High anti-dsDNA antibodies, low C3 or C4 allowed | High anti-dsDNA antibodies, low C3 or C4 allowed | High anti-dsDNA antibodies, low C3 or C4 allowed | **-** | **-** |
| **GCs** | Not allowed | Prednisolone ≤5mg/day or equivalent | Not allowed | Prednisolone ≤5mg/day or equivalent | Prednisolone ≤7.5mg/day or equivalent | Prednisolone ≤7.5 mg/day or equivalent | Not allowed | **-** | **-** |
| **DMARDs/ biologic treatments** | HCQ only | Accepted^c^ | HCQ only | Accepted^c^ | Accepted^c^ | Accepted^c^ | HCQ only | **-** | **-** |

^a^renal, CNS, cardiopulmonary, vasculitis, fever; no heamolitic anemia, or GI involvement; ^b ^Only one manifestation of: rash, alopecia, mucosal ulcers, pleurisy, pericarditis, fever, thrombocytopenia, leukopenia; ^c^maintenance dose

SLE, systemic lupus erythematosus; DORIS, Definition of Remission In SLE; APLC, Asia-Pacific Lupus Collaboration; LLDAS, Lupus Low Disease Activity Status; LCTC, Lupus Clinical Trials Consortium; cSLEDAI-2k, (clinical) Systemic Lupus Erythematosus Disease Activity Score-2000; BILAG, British Isles Lupus Assessment Group; PGA, Physician Global Assessment; DMARDs, Disease Modifying Anti-Rheumatic Drugs; HCQ, hydroxychloroquine; dsDNA antibodies, Anti-double-stranded DNA antibodies; GCs, glucocorticoids; GI, gastrointestinal.

**Supplementary Table S2. Distribution of jSLE patient visits**

| **Number of visits per patient** | **Proportion of patients (%)** |
| --- | --- |
| **1** | 63.6 |
| **2** | 11.4 |
| **3** | 14.7 |
| **4** | 4.6 |
| **5** | 2.3 |
| **6** | 3.4 |

jSLE, Juvenile Systemic Lupus Erythematosus

**Supplementary Table S3. Cross-sectional analysis assessing serum IFN-α2 levels in jSLE patients, JIA patients an HCs**

|  | **jSLE** **(n=88)** | **JIA** **(n=35)** | **HCs** **(n=28)** | **Adjusted p-values*** |  |  |
| --- | --- | --- | --- | --- | --- | --- |
|  |  |  |  | **jSLE vs. JIA** | **jSLE vs. HCs** | **JIA vs. HCs** |
| **IFN-α2 levels, fg/mL, median** **[IQR]** | 587 [11-2,744] | 7 [3-236] | 29 [3-277] | **0.0017** | **0.017** | 0.581 |

*Kruskal-Wallis multiple comparison p-value followed by Dunn’s post hoc tests with Benjamini-Hochberg p-value correction method. Post-hoc adjusted p-values are reported. N=number of patients/samples. IFN-α2, Interferon-alpha2; jSLE, Juvenile Systemic Lupus Erythematosus; JIA, Juvenile Idiopathic Arthritis; HCs, Healthy Controls; vs., versus; fg/mL, femtograms per milliliter; IQR, interquartile range.

**Supplementary Table S4. Cross-sectional analysis assessing serum IFN-α2 levels in JIA subgroups**

|  | **Oligo JIA** **(n=11)** | **Poly JIA** **(n=16)** | **Psoriatic JIA** **(n=3)** | **SJIA** **(n=5)** | **Adjusted p-values*** |  |  |  |  |  |
| --- | --- | --- | --- | --- | --- | --- | --- | --- | --- | --- |
|  |  |  |  |  | **Oligo vs. Poly JIA** | **Oligo vs. Psoriatic JIA** | **Oligo vs. SJIA** | **Poly vs. Psoriatic JIA** | **Poly vs. SJIA** | **Psoriatic vs. SJIA** |
| **IFN-α2 levels, fg/mL, median** **[IQR]** | 3 [10-204] | 3 [3-661] | 128 [66-34,737] | 3 [3-7] | 1.000 | 1.000 | 1.000 | 1.000 | 0.750 | 0.472 |

*Kruskal-Wallis multiple comparison p-value followed by Dunn’s post hoc tests with Benjamini-Hochberg p-value correction method. Post-hoc adjusted p-values are reported. N=number of patients/samples. IFN-α2, interferon-alfa2; JIA, Juvenile Idiopathic Arthritis; Oligo, oligoarticular; Poly, polyarticular; SJIA, Systemic Juvenile Idiopathic Arthritis; vs., versus; fg/mL, femtograms per milliliter; IQR, interquartile range.

**Supplementary Table S5. Cross-sectional analysis assessing serum IFN-α2 levels in different patient groups and healthy controls according to participant sex** 

|  | **JSLE (n=88)** |  | **JIA (n=35)** |  | **HCs (n=28)** |  |
| --- | --- | --- | --- | --- | --- | --- |
|  | **Female** **(n=71)** | **Male** **(n=17)** | **Female** **(n=24)** | **Male** **(n=11)** | **Female** **(n=23)** | **Male** **(n=5)** |
| **IFN-α2 levels, fg/mL, median** **[IQR]** | 741 [11-2,845] | 145 [13-1,037] | 14 [3-353] | 3 [3-15] | 25 [3-269] | 110 [17-270] |
| **P-value*** | 0.678 |  | 0.322 |  | 0.626 |  |

*Mann-Whitney test with continuity correction. N=number of patients/samples. IFN-α2, interferon-alfa2; jSLE, juvenile systemic lupus erythematosus; JIA, juvenile idiopathic arthritis; HCs, healthy controls; fg/mL, femtograms per milliliter; IQR, interquartile range.

**Supplementary Table S6. Cross-sectional analysis assessing serum IFN-α2 levels and disease activity states in jSLE patients according to ethnicity**

|  | **White** **(n=35)** | **Black African/Caribbean** **(n=16)** | **Asian** **(n=29)** | **Adjusted p-values*** |  |  |
| --- | --- | --- | --- | --- | --- | --- |
|  |  |  |  | **White vs. Black African/Caribbean** | **Asian vs. White** | **Black African/Caribbean vs. Asian** |
| **IFN-α2 levels, fg/mL, median** **[IQR]** | 134 [3-1,255] | 1,326 [647-7,503] | 570 [3- 2,130] | **0.028** | 0.390 | 0.102 |
| **Disease activity states** Active/intermediate LDAS Remission | 11 16 8 | 8 5 3 | 12 14 3 | 0.406 | 0.716 | 0.906 |

*Kruskal-Wallis multiple comparison p-value followed by Dunn’s post hoc tests with Benjamini-Hochberg p-value correction method. Post-hoc adjusted p-values are reported. Ethnicity data are not available for 8 patients. N=number of patients/samples. IFN-α2, interferon-alfa2; jSLE, juvenile systemic lupus erythematosus; vs., versus; fg/mL, femtograms per milliliter; IQR, interquartile range; LDAS, low disease activity state

**Supplementary Table S7. Cross-sectional analysis assessing serum IFN-α2 levels in jSLE patients according to prednisolone dosage** 

|  | **No prednisolone** **(n=47)** | **Low dose prednisolone dose (≤ 7.5 mg/day, n=30)** | **Medium–high dose prednisolone (> 7.5 mg/day, n=11)** | **Adjusted p-values*** |  |  |
| --- | --- | --- | --- | --- | --- | --- |
|  |  |  |  | **No prednisolone vs.** **Low dose prednisolone (≤ 7.5 mg/day)** | **No prednisolone vs.** **Medium-high dose prednisolone (> 7.5 mg/day)** | **Low dose prednisolone (≤ 7.5 mg/day) vs.** **Medium-high dose prednisolone (> 7.5 mg/day)** |
| **IFN-α2 levels, fg/mL, median** **[IQR]** | 409 [3-1,818] | 702 [28-1,994] | 4,224 [123-19,127] | 0.463 | 0.382 | 0.504 |

*Kruskal-Wallis multiple comparison p-value followed by Dunn’s post hoc tests with Benjamini-Hochberg p-value correction method. Post-hoc adjusted p-values are reported. N=number of patients/samples. IFN-α2, interferon-alfa2; jSLE, juvenile systemic lupus erythematosus; mg, milligrams; vs., versus; fg/mL, femtograms per milliliter; IQR, interquartile range

**Supplementary Table S8. Cross-sectional analysis comparing serum IFN-α2 levels between jSLE sub-groups, and jSLE patients in remission with HCs**

|  | **jSLE (n=88)** |  |  | **HCs (n=28)** | **Adjusted p-values*** |  |  |  |
| --- | --- | --- | --- | --- | --- | --- | --- | --- |
|  | **Active or intermediate disease activity** **(n=35)** | **LDAS** **(n=39)** | **Remission** **(n=14)** |  | **Active or intermediate vs.** **LDAS** | **Active or intermediate vs. remission** | **LDAS vs. remission** | **Remission vs. HCs** |
| **IFN-α2 levels, fg/mL, median** **[IQR]** | 3,185 [48-13,703] | 571 [57-1,310] | 271 [3-56] | 29 [3-277] | **0.04** | **<0.001** | 0.05 | 0.37^**#**^ |

*Kruskal-Wallis multiple comparison p-value followed by Dunn’s post hoc tests with Benjamini-Hochberg p-value correction method. Post-hoc adjusted p-values are reported. ^#^Mann-Whitney test with continuity correction. N=number of patients/samples. IFN-α2, Interferon-alpha2; jSLE, Juvenile Systemic Lupus Erythematosus; HCs, Healthy Controls; LDAS, Low Disease Activity State; vs., versus; fg/mL, femtograms per milliliter; IQR, interquartile range.

**Supplementary Table S9. Cross-sectional analysis assessing serum IFN-α2 levels and flare occurrence at 6 and 12 months in jSLE patients**

|  | **Flare at 6 months** **(n=66)** |  | **Flare at 12 months** **(n=66)** |  |
| --- | --- | --- | --- | --- |
|  | Yes (n=27) | No (n=39) | Yes (n=38) | No (n=28) |
| **IFN-α2 levels, fg/mL, median** **[IQR]** | 1,297 [448-7,365] | 409 [46-1,410] | 1,189 [335-7,601] | 222 [46-1,283] |
| **P-value*** | 0.058 |  | 0.036 |  |

*Mann-Whitney test with continuity correction. Data on flare occurrence are not available in 22 patients. N=number of patients/samples. IFN-α2, Interferon-alpha2; jSLE, Juvenile Systemic Lupus Erythematosus; fg/mL, femtograms per milliliter; IQR, interquartile range

**Supplementary Table S10. Longitudinal analysis comparing serum IFN-α2 levels in jSLE patients stratified for disease activity state**

| **Outcome variable** | **Patients with high/intermediate disease activity state** **(n=71)** | **Patients in LDAS** **(n=65)** | **Patients in remission (n=23)** |
| --- | --- | --- | --- |
| Mean (SD), fg/mL | 7,653 (14,282) | 2,057 (6,378) | 275 (489) |
| Median (IQR), fg/mL | 1,583 (100-7,850) | 228 (3-1,278) | 11 (3-291) |
| Min-Max, fg/mL | 3-88,064 | 3-41,587 | 3-1,588 |
| GLMM p-value (fg/mL) | NA (reference group) | 0.0993 (-3,096) | 0.0898 (-4,266) |

N=number of measurements. IFN-α2, Interferon-alpha2; jSLE, Juvenile Systemic Lupus Erythematosus; LDAS, Low Disease Activity Status; SD, Standard Deviation; fg/mL, femtograms per milliliter; IQR, interquartile range; Min, minimum; Max, maximum; GLMM, generalised linear mixed model; NA, not applicable

**Supplementary Table S11. Longitudinal analysis comparing time to flare (within 6 and 12 months) in all jSLE patients with normal and abnormal IFN-α2 levels**

| **Outcome variable** | **All patient visits (n=159)** | **Patients with abnormal IFN-α2 (n=66)** | **Patients with normal IFN-α2** **(n=93)** | **Cox model with random effects** **HR (95% CI)** | **P-values** |
| --- | --- | --- | --- | --- | --- |
| **Time to flare within 6 months of IFN-α2 quantification (days)** |  |  |  |  |  |
| Mean (SD) | 110.0 (40.0) | 94.3 (35.9) | 130.4 (36.7) | 2.1 (1.0-4.2) | **0.041*** |
| Median (IQR) | 115.5 (80.5,133.8) | 91.0 (70.0, 126.0) | 128.0 (119.0, 168.0) |  |  |
| Min, Max | 34.0, 168.0 | 70.0, 154.0 | 34.0, 168.0 |  |  |
| Flare, n (%) No flare, n (%) NA, n (%) | 30 (39) 47 (61) 82 | 17 (51.5) 16 (48.5) 33 | 13 (29.5) 31 (70.5) 49 |  | 0.062^#^ |
| **Time to flare within 12 months of IFN-α2 quantification (days)** |  |  |  |  |  |
| Mean (SD) | 142.1 (65.2) | 126.7 (70.9) | 159.1 (55.2) | 1.8 (1.0-3.1) | 0.052* |
| Median (IQR) | 142.1 (105.0, 187.2) | 108.5 (77.0, 152.0) | 168.0 (126.0, 192.2) |  |  |
| Min, Max | 34.0, 308.0 | 36.0, 301.0 | 34.0, 308.0 |  |  |
| Flare, n (%) No flare, n (%) NA, n | 42 (54.5) 35 (45.5) 82 | 22 (66.7) 11 (33.3) 33 | 20 (45.5) 24 (55.5) 49 |  | 0.071^**#**^ |

N in the column headers=number of measurements; n in the rows=number of flare events. JSLE, juvenile systemic lupus erythematosus; IFN-α2, interferon-alpha2; HR, hazard ratio; CI, confidence interval; SD, standard deviation; IQR, interquartile range; Min, minimum; Max, maximum; n, number; NA, not available

**Supplementary Table S12. Longitudinal analyses investigating the ability of IFN-α2 and standard clinical biomarkers to predict time to flare in jSLE**

| **Univariable analyses** | **Cox model with random effects** **HR (95% CI)** | **Cox model with random effects** **P-value** |
| --- | --- | --- |
| **IFN-α2 levels, fg/mL** (n=148, 79 events) Normal (n=89) Abnormal (n=59) | 1.80 (0.93, 3.46) | 0.079 |
| **Anti-dsDNA abs, IU/mL** (n=145, 76 events) <20 (n=88) ≥20 (n=57) | 0.96 (0.44, 2.10) | 0.93 |
| **C3 levels, g/dL** (n=147, 78 events) ≥0.90 (n=82) <0.90 (n=65) | 0.70 (0.34, 1.44) | 0.34 |
| **ESR, mm/h** (n= 146, 78 events) Normal <10 (n=67) Mildly to mod raised 10-50 (n=56) High >50 (n=23) | 0.89 (0.46, 1.71) 1.51 (0.53, 4.27) | 0.72 0.44 |
| **Multivariable analysis** (n=143, 75 events) |  |  |
| **IFN-α2 levels, fg/mL** Normal Abnormal | 1.78 (0.85-3.75) | 0.13 |
| **Anti-dsDNA abs, IU/mL** <20 ≥20 | 0.92 (0.36-2.37) | 0.86 |
| **C3 levels, g/dL** ≥0.90 <0.90 | 0.62 (0.28-1.38) | 0.24 |
| **ESR, mm/h** Normal <10 Mildly to mod raised 10-50 High >50 | 0.76 (0.37-1.58) 1.55 (0.45-5.31) | 0.47 0.49 |

N=number of measurements for which laboratory data were available. IFN-α2, interferon-alpha2; jSLE, juvenile systemic lupus erythematosus; OR, odd ratio; CI, confidence interval; SD, standard deviation; fg/mL, femtograms per milliliter; anti-dsDNA abs, anti-double stranded DNA antibodies; IU/mL, International Units per milliliter; g/dL, grams per deciliter; ESR, erythrocyte sedimentation rate
